# Supplementary figures and images for: Cortical oscillations support sampling-based computations in spiking neural networks
Source: PLoS Comput Biol. 2022 Mar 24;18(3):e1009753. doi: 10.1371/journal.pcbi.1009753 (PMC8947809; doi:10.1371/journal.pcbi.1009753)

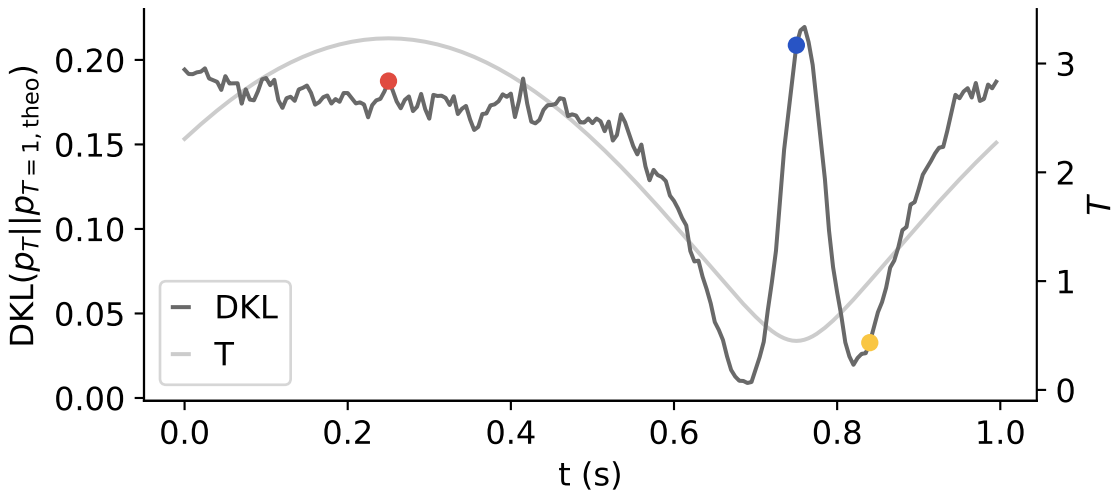

Supplement: S1 Fig — Time course of the Kullback-Leibler divergence to the target distribution together with the time course of the temperature demonstrated at the network in Fig 2. The KL-divergence is high for both high temperatures (red dot, quasi-uniform distribution) and low temperatures (blue dot, quasi-single state distribution), indicating that the distributions at these temperatures differ. The divergence is small at the two crossings of T = 1, indicating high fidelity representations. The yellow dot indicates the time of the readout. (PDF) [file pcbi.1009753.s001.pdf]

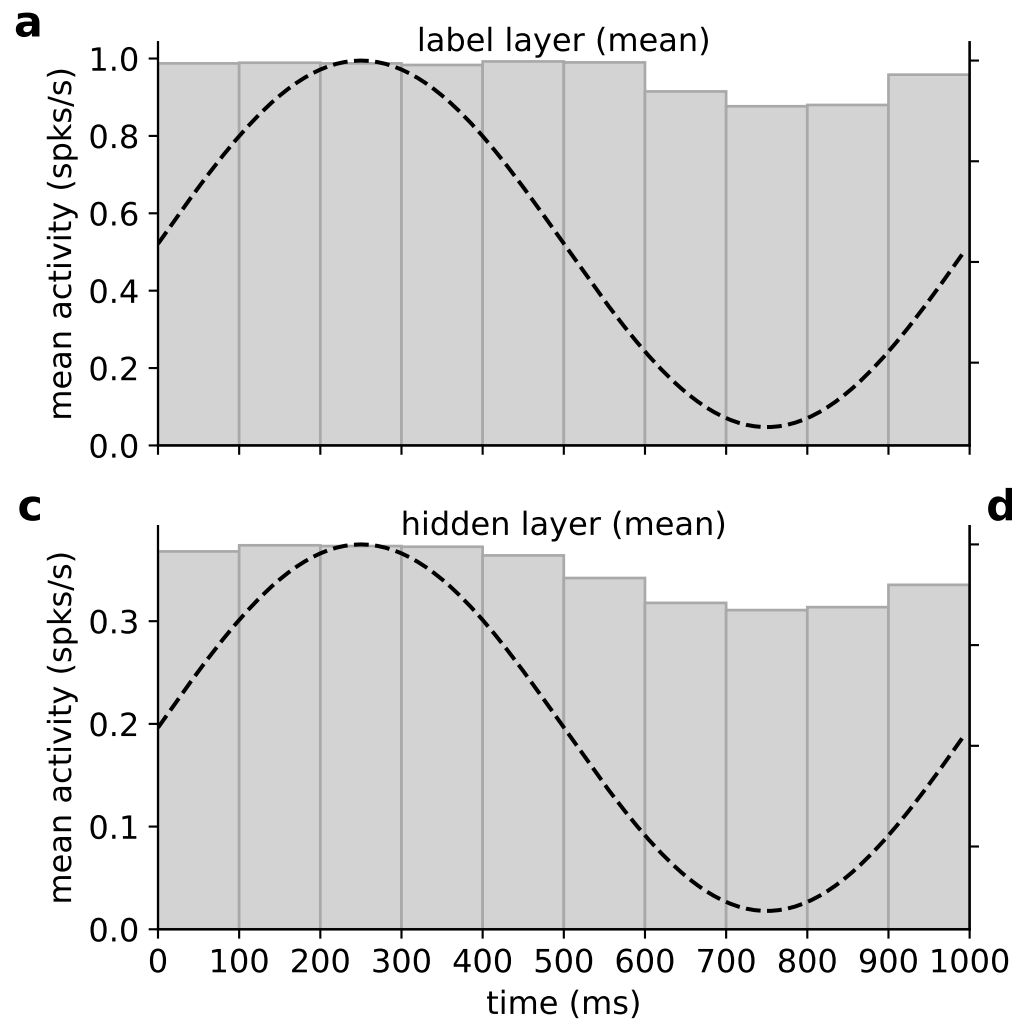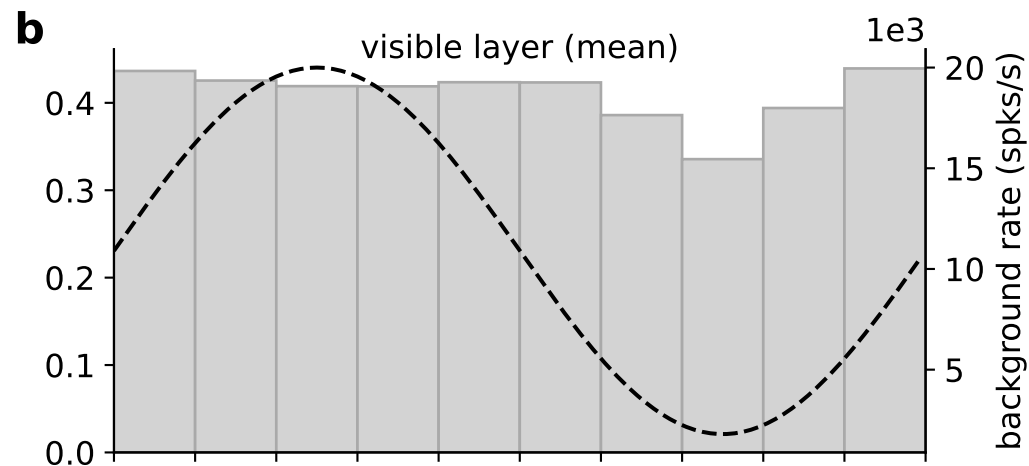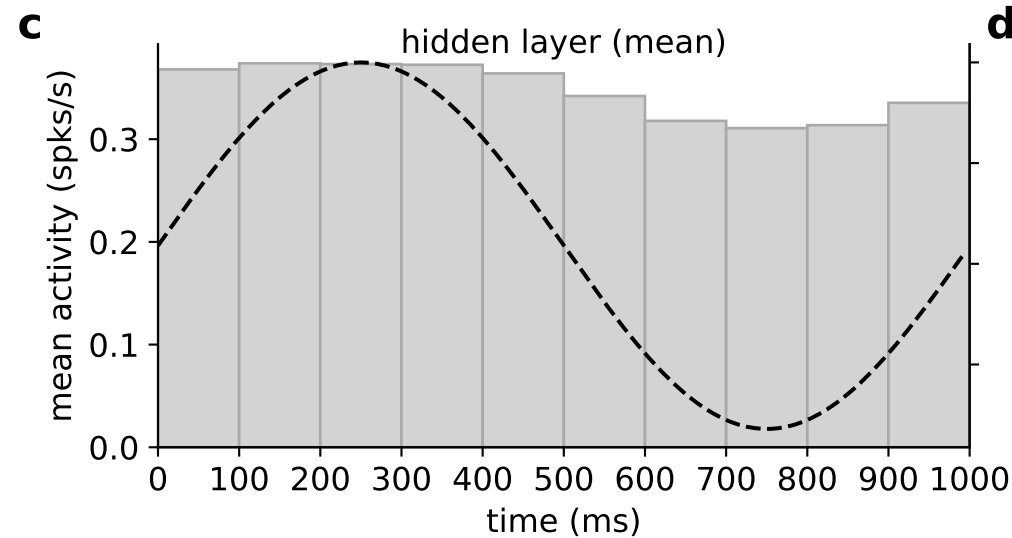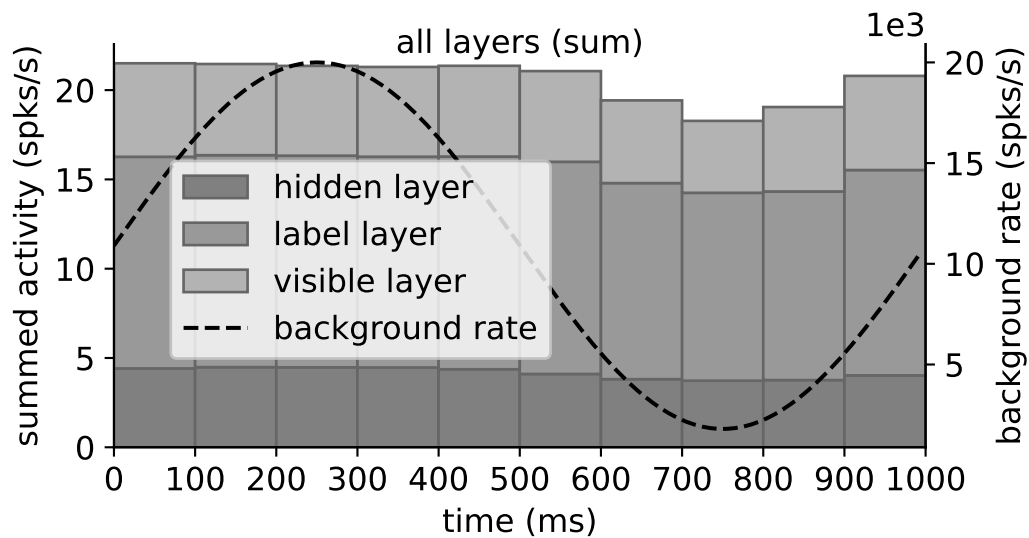

Supplement: S2 Fig — (a-c) Spike activity in label, visible and hidden layer of the NORB network in Fig 3 as a function of the phase of the background oscillation. The mean firing rate per neuron oscillates in all three layers in phase with the background. (PDF) [file pcbi.1009753.s002.pdf]

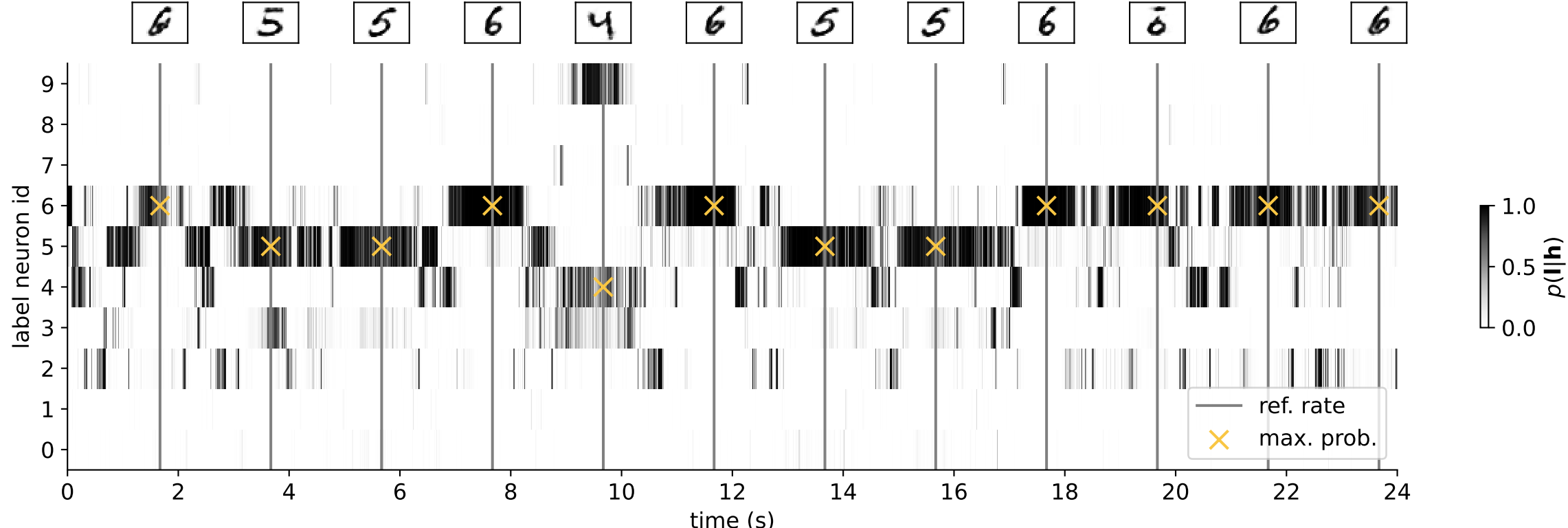

Supplement: S3 Fig — Exemplary time course of the inferred activity per label neuron over time (lower plot) and the associated state of the visible layer (top bar) of the MNIST network in Fig 4. Spike probability is high and unique during the low activity phases (around the T = 1 readout, vertical lines) and lower and distributed over several labels during the high activity phases. The network is typically in a stable response state for a certain time window around the readout. The length of this time window depends on the depth of the modes. (PDF) [file pcbi.1009753.s003.pdf]

**a**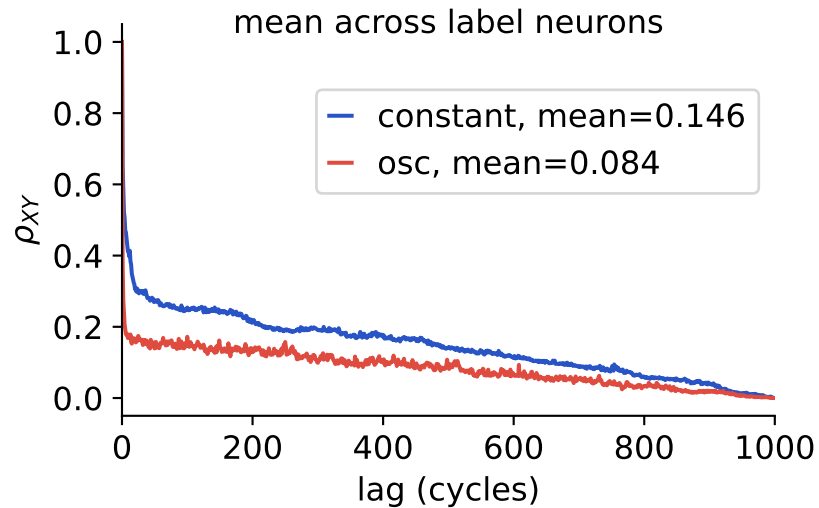**b**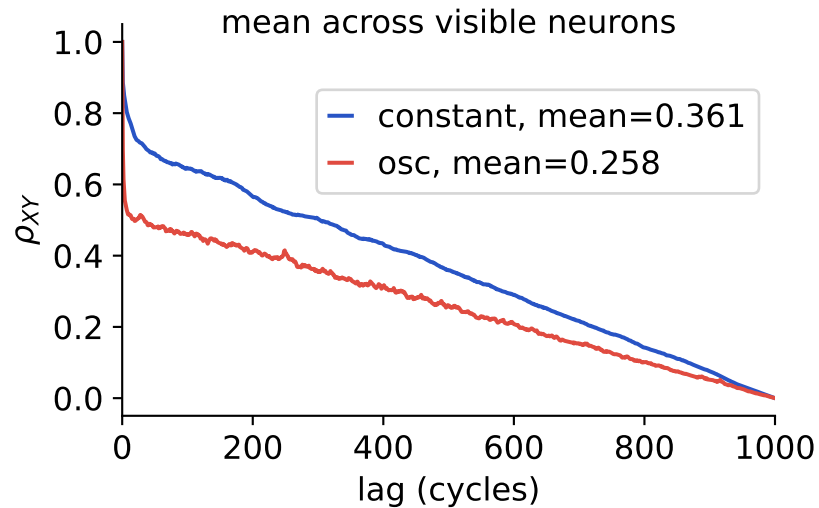

Supplement: S4 Fig — (a) Mean Pearson autocorrelation coefficient calculated from the inferred spike probability of the label layer neurons of the MNIST network in Fig 4—for oscillating background (red) and constant background at T = 1 (blue). (b) Same as (a), for the visible neurons. Autocorrelation is reduced more quickly for the oscillating setup, leading to a smaller area under the curve, indicating faster mixing. (PDF) [file pcbi.1009753.s004.pdf]

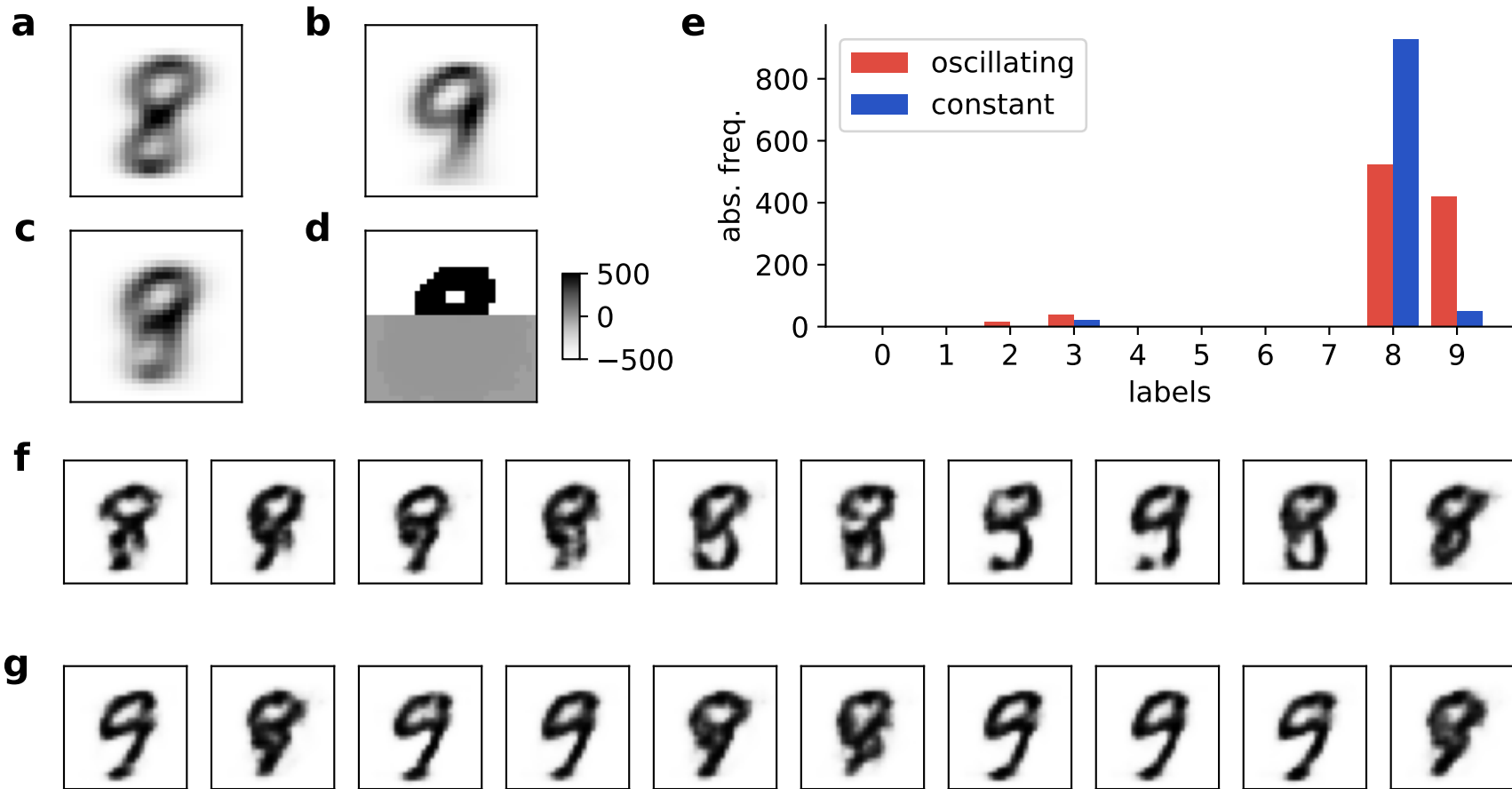

Supplement: S5 Fig — Superposition of the first 5421 images of class 8 (a) and class 9 (b) of the MNIST training data set. (c) Superposition of images in a and b. (d) Biases of the network to clamp visible layer to the upper part of the image in c and emulate an ambiguous input. (e) Distribution over the inferred labels of the MNIST network from Fig 4 in a 100-cycles run averaged over ten random seeds. The imprinted labels 8 and 9 dominate the distribution—the posterior distribution—illustrating the uncertainty of the input. With oscillating background input, the distribution is more balanced. Thus, oscillations can help in inference tasks. Note that the network simultaneously completes the lower part of the ambiguous input image in the visible layer—shown as the inferred visible layer activity for constant background in (f) and oscillating background in (g). (PDF) [file pcbi.1009753.s005.pdf]

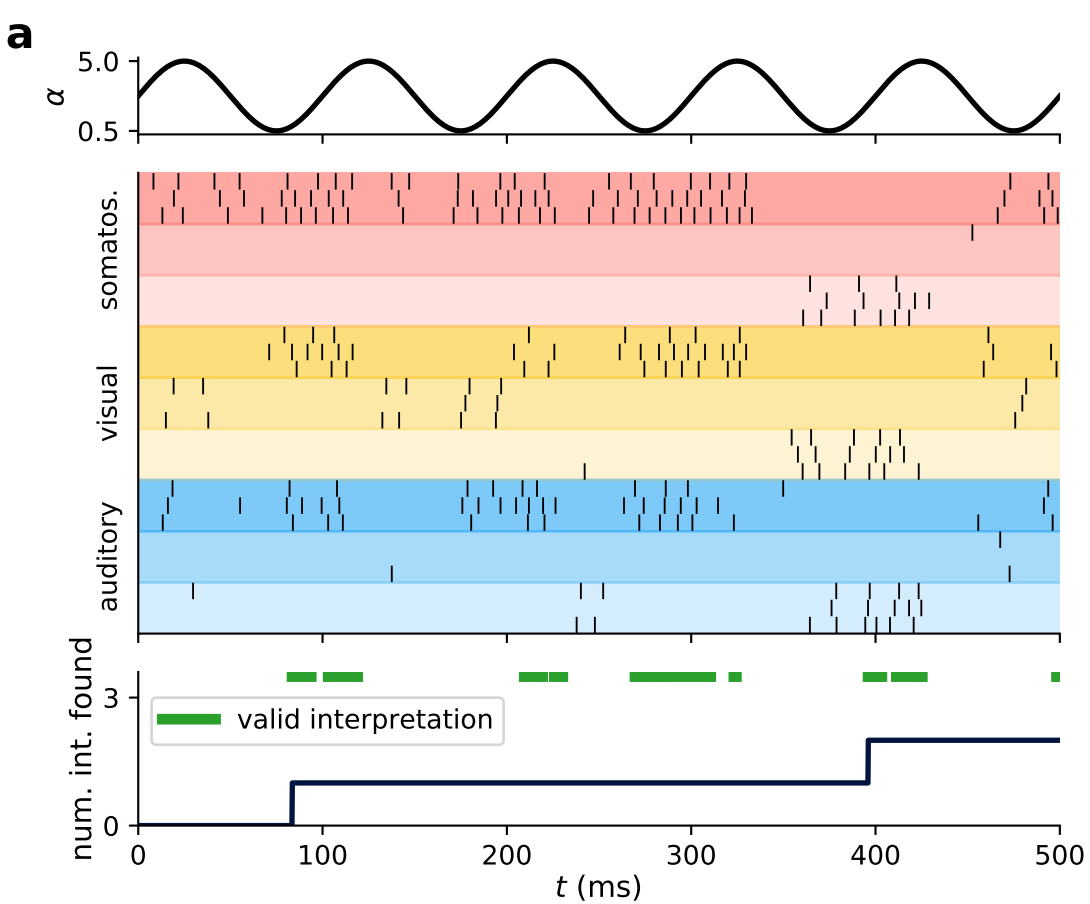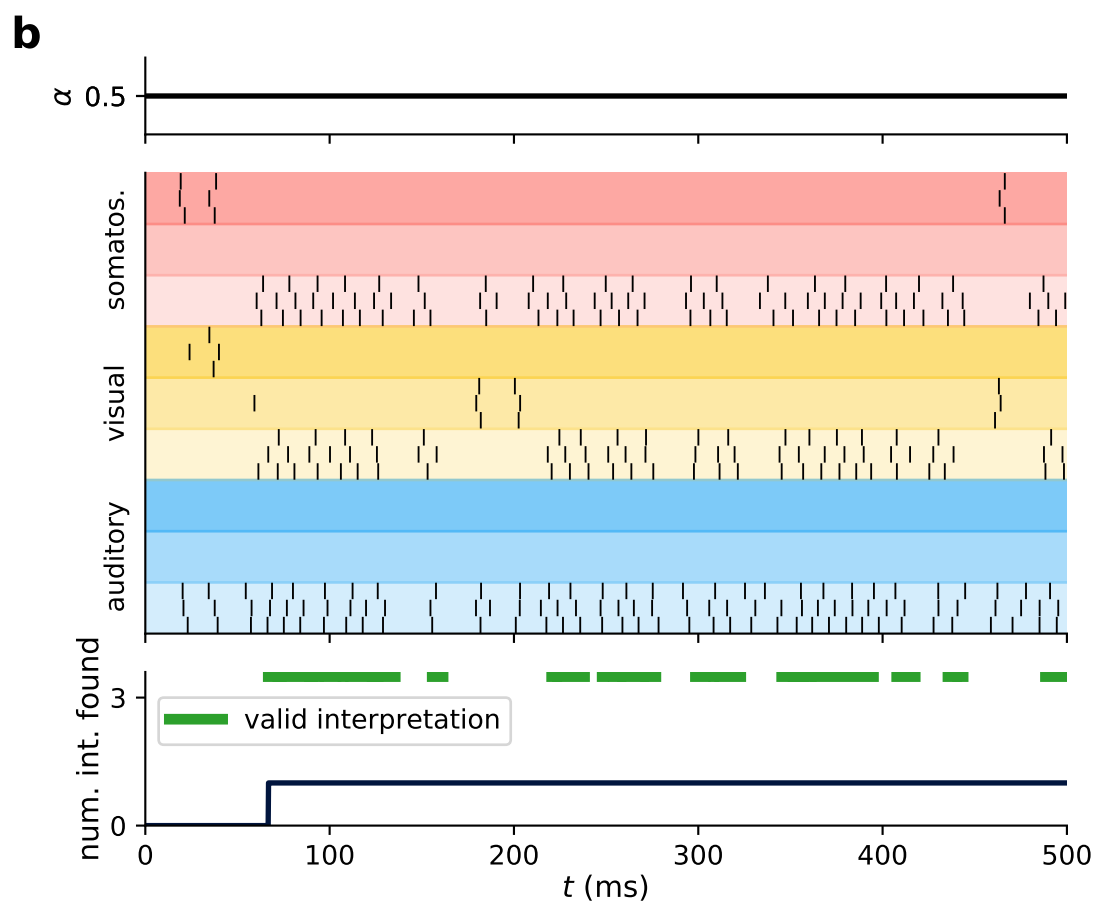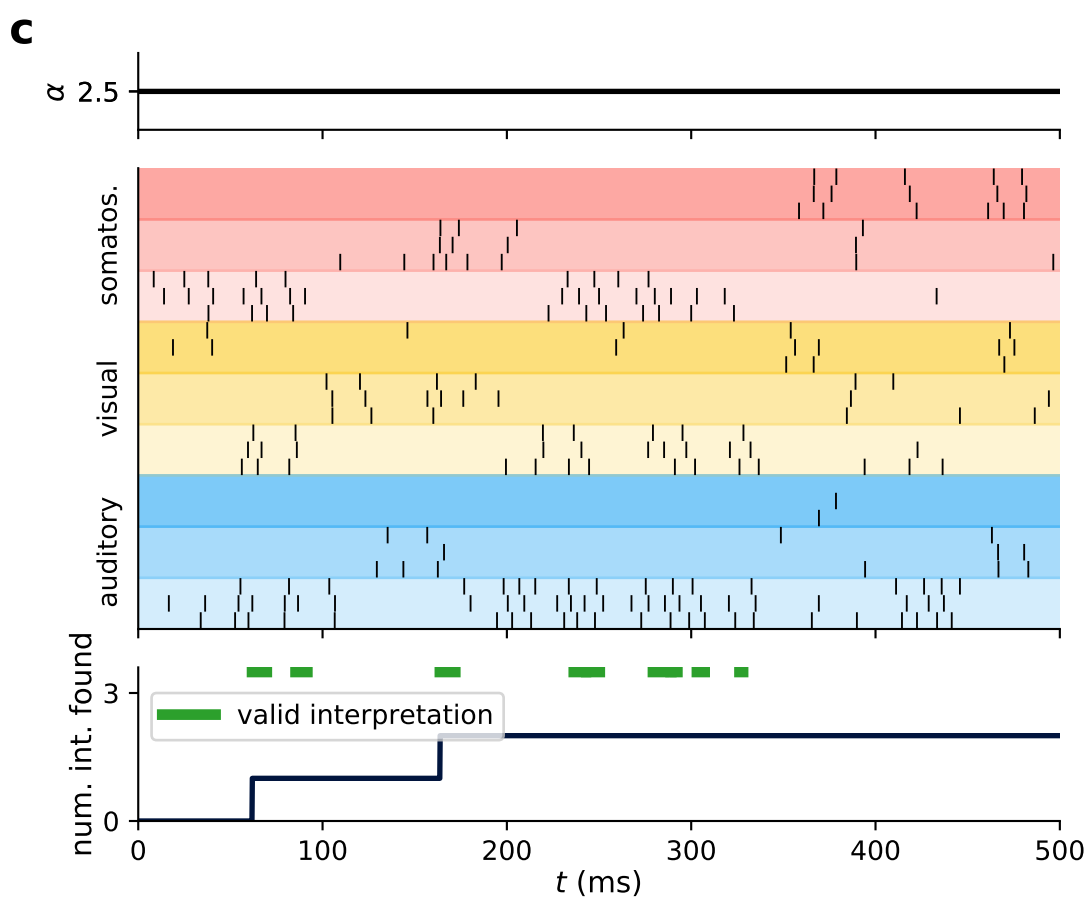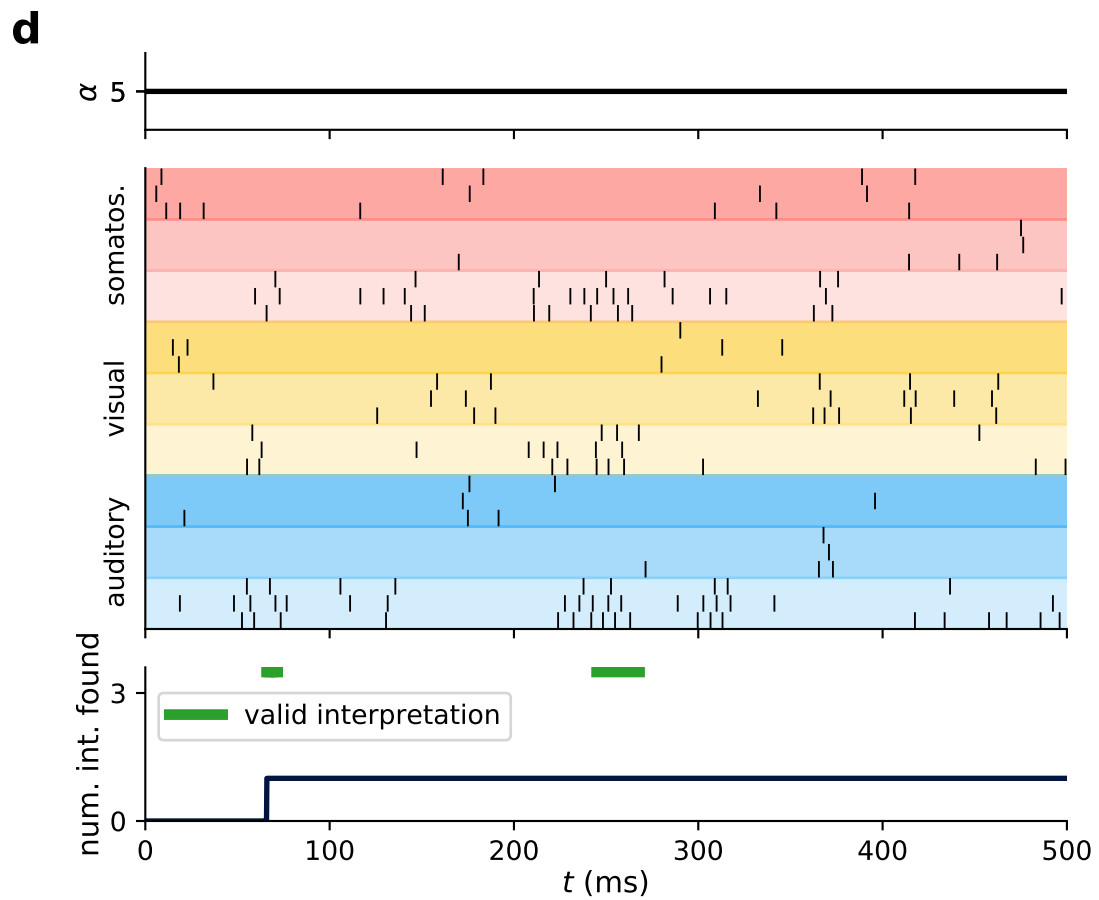

Supplement: S6 Fig — (a) Sample activity for oscillating background input (see Fig 6b for details). (b-d) Sample activity for constant background input with α ∈ {0.5, 2.5, 5}. (PDF) [file pcbi.1009753.s006.pdf]

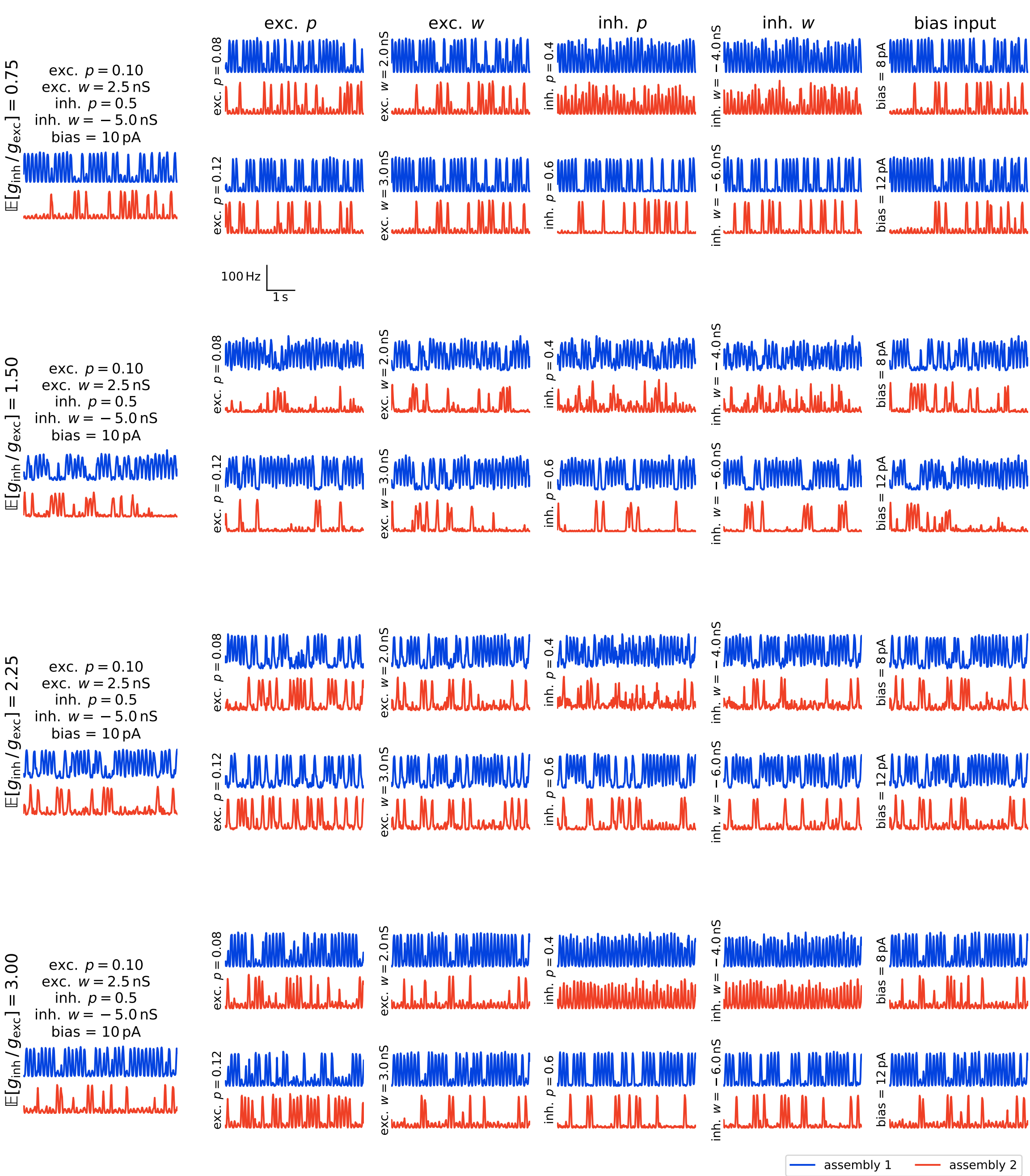

Supplement: S7 Fig — For four values of E[ginh/gexc] (rows, as in Fig 7b), we show activity for systematic variations of the model parameters. In each row, the plot on the left corresponds to the plot in Fig 7b with the base parameters indicated. On the right, each column shows sample activity when one of these parameters (see column title) is varied by multiplying it with 0.8 (top panels within each row) or 1.2 (bottom panels within each row). Variations of the inhibition have the largest impact on the model behavior, with decreased inhibition leading to simultaneous activity of both assemblies in some cases. (PDF) [file pcbi.1009753.s007.pdf]

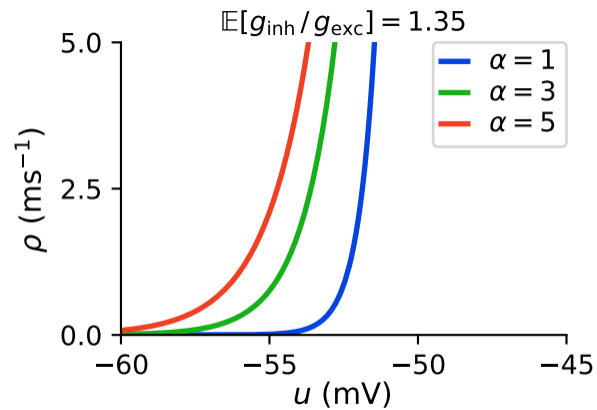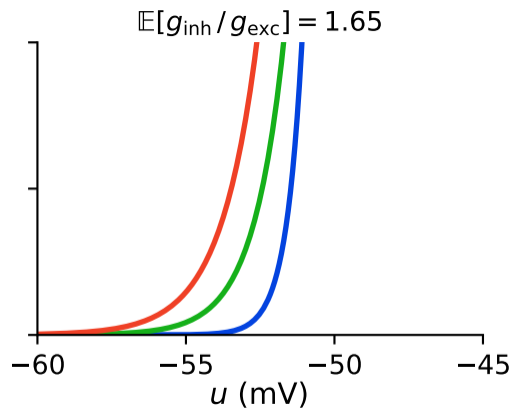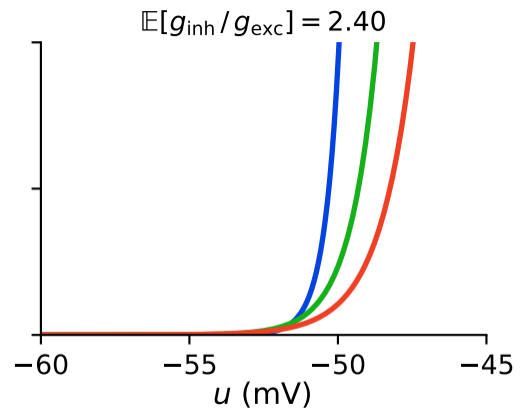

Supplement: S8 Fig — Firing intensity behavior of individual neurons determined by fitting stochastic models (as in Fig 9b) drastically changes as the mean background conductance ratio E[ginh/gexc] is increased. (PDF) [file pcbi.1009753.s008.pdf]
