# Supplementary material for: Cortical oscillations support sampling-based computations in spiking neural networks
Source: PLoS Comput Biol. 2022 Mar 24;18(3):e1009753. doi: 10.1371/journal.pcbi.1009753 (PMC8947809; doi:10.1371/journal.pcbi.1009753)
Supplement: Attachment — Submitted filename: response_to_reviewers.pdf [file pcbi.1009753.s009.pdf]

Author response to reviewer comments

First of all, we wish to thank the reviewers for their encouraging feedback, as well as for their detailed comments and suggestions. Indeed, we feel that this interaction has led to a significantly improved manuscript. We hope to have clarified all remaining questions in our point-by-point reply below, as well as in the revised manuscript. For easier navigation, we numbered the individual points addressed by the reviewers in the margins of the reply letter. The version of the manuscript which includes highlighted changes also references these numbers in the margins.

Reviewer #1

Big-picture comments (these are suggestions; I would be happy to recommend acceptance without them being addressed):

- The manuscript is long with 9 Figures in the main text. The paper might ultimately have more impact if it were streamlined (with Figures merged or pushed to supplementary).

[#1]

> We agree with this suggestion. We streamlined the paper by merging the illustrative Fig. 2 with Fig. 3, merging Figs. 6 and 7, and moving some parts of the original Figs. 3, 6, and 9 to supplementary figures. The main text now has 7 figures instead of 9, with fewer panels than before.

- The section on matching experimental data (Fig. 9) is relatively short. Adding more here would be great (but I am well aware this may not be possible).

[#2]

> (Note: this is now Fig. 7.) We added some more details regarding the model and an intuitive description of the behavior (l. 449 and below) as well as a schematic figure (Fig. 7a) to clarify this part of the paper.

Minor comments

- "we unify two individually well-studied, but previously unlinked aspects of cortical dynamics under a common normative framework: probabilistic inference and cortical oscillations." Given Aitchison and Lengyel (2016), Echeveste et al. (2021) and Savin et al. (2014), the claim that these are "previously unlinked" is too strong. But these papers are not novelty-destroying as e.g. they use rates rather than spikes.

[#3]

> We thank the reviewer for pointing this out. We have worded our claim more carefully now (l. 43).

- Savin (2014) would seem to be the most relevant prior art, and it is currently a bit buried in the "Related work". It should be disucssed earlier in the Introduction and more extensively.

[#4]

> Savin et al. (2014) is now introduced in the Introduction (l. 56). We furthermore expanded the corresponding part of the Discussion (l. 533).

- Eq. 1+2 appear to ignore correlations in the presynaptic inputs. But I buy the overall logic.

[#5]

> This is, of course, correct. Therefore, we added a corresponding sentence to make this explicit (l. 97).

- "Upon introducing a firing threshold, some portion of the free membrane potential will lie above it" some portion of the membrane potential *probability density* lies above the threshold.

[#6]

> We added the missing term (l. 105).

- The figures are occasionally described in a cursory fashion, e.g. "Overall, the best performance was achieved in the slow-wave regime (Fig. 5c-g)".

[#7]

> We improved the text by more closely linking it to the figures (see, e.g., l. 237 to 246 for the discussion of Fig. 4 (formerly Fig. 5)). See also #42 below.

- Could do with a bit more clarity about exactly what equations are actually being run for the simulations (i.e. was it really an LIF in the sims, or was there a Boltzmann Machine with a variable temperature?). If nothing else, it is necessary to state the equations for the LIF, and tell us specifically how the background activity was implemented in the main text.

[#8]

We thank the reviewer for pointing out this important issue. We have now clarified at multiple locations throughout the text that all of our simulations use LIF neurons (e.g., l. 74, l. 124, l. 190, captions of Fig. 3 and 4). The neuron models themselves are described in detail in the first few sections of the Methods.

- The experiments for the conductance based network appears considerably more simplistic than those for the current based network. Can the authors comment on the difficulties?

[#9]

Instead of focusing on a specific task like image generation as in the current-based case with a particular set of assumptions such as unbiased sampling (which in principle poses no problem even in the conductance-based case), we chose to focus on more traditionally considered tasks regarding perception. This has the added benefit of highlighting that the proposed effect of background input oscillations holds regardless of the specific model (and model details, as we also show using conductance-based models). To clarify this, we expanded the discussion of the difference between the two types of models and their motivation (l. 260 and l. 436).

- The use of insets in plots should be minimized as it is very difficult to e.g. write on axis labels.

[#10]

We removed the insets in Figs. 2 and 4 and partially removed them in Fig. 7.

- More careful referencing to the relevant part of Methods would be appreciated.

[#11]

We changed the references to the Methods by including the specific subsection to which we are referring – see, e.g., l. 96.

Reviewer #2

Summary

This manuscript presents the intriguing and promising proposal that cortical oscillations may serve the functional role of speeding up computational dynamics, especially the transitions between different modes in multimodal distributions. The paper shows how excitatory and inhibitory background firing rates act together to determine slope and operating point of the response functions of current-based and conductance-based LIF neurons. It interprets the slope of the response function as a computational temperature on the distribution that the cortical states are sampled from. It then implements the idea of rhythmic changes in the background activity in several spiking network models and illustrates the effect visually and using multiple statistics. While the idea is novel and interesting, I have several major concerns about both implementation and exposition.

1. As far as I know, cortical oscillations are commonly believed to reflect changes in collective mean activity of neurons, not the slope of the input-output function of individual neurons. However, in the authors' proposal, the mean is kept constant so it's not clear to me that classical measurements of oscillations would be able to measure them in the authors' model. Is this correct? Furthermore, recent work (Engels et al. Science 2016) has found that high activity states were associated with high behavioral performance which appears to be in contradiction with the authors' idea that high (background) rates are associated with a high temperature?

[#12]

Our model is also based on the assumption that oscillations change the mean activity (although it is known that this coupling is sometimes not very strong, see, e.g., Csicsvari et al., 2003, Neuron), which is why the background input rates increase and decrease over each cycle. We have shown that this changes the input-output function of neurons receiving such an input (see Eq. 3). Whether this results in changes in the mean neuronal activities and to what extent depends on several factors such as network architecture and parameters. As an example, we plotted the changes of the mean activity over each oscillatory cycle for the

model used in Fig. 3 in the new Fig. S1 in the *Supplementary Material*. Here, while there are some phase-dependent changes, the differences are rather small. On the other hand, the model of place-cell flickering shows pronounced changes in the mean firing activity (Fig. 7c), particularly for the parameter regions we found to match the experimental data. Thus, depending on the precise model details, our model is well-suited to reproduce different experimental findings, including large phase-dependent rate changes.

Engel et al. (2016) investigated the processing in local cortical circuits (in monkey V4), which show high firing activity (on-state) or low activity (off-state). They showed that a change of the input conditions was detected with higher accuracy if the precise timing of the change coincided with an on-state within the cortical network. This finding fits well with our results. According to our model, a high overall rate results in a high temperature, allowing networks to move around in their state space and switch between alternative explanations. Low firing rates result in a low temperature, with which it is hard to change an interpretation – which is required when a change of the input signal should be detected. Thus, if a change occurs in a high activity phase, it should be much easier to detect as it is easier for the network to switch to an alternative state (i.e., explanation of the inputs) in this phase. The main difference between these findings and our model is that we use a gradual (sinusoidal) scheme to alternate between high and low activity phases, whereas the data by Engel et al. suggests that the changes occur abruptly. However, our model could also implement such a scheme, which we strongly expect to have the same effect. We added a discussion of this point to the text (l. 640).

2. How exactly does Eq 4 establish the relationship between ensemble temp and background firing rate? One is the slope of the input-output function, the other the scaling of the joint probability function over all states z. Please elaborate.

[#13]

The form of the neuronal response function (Eq. 3) and the temperature-dependent conditional probability of a single neuron being in the state $z = 1$ (Eq. 6) are identical, thus allowing us to relate the slope β of the activation function to the ensemble temperature T (Eq. 7). We added more details regarding this derivation in the text to clarify this relationship (see e.g. l. 120, l. 135, l. 138, l. 167).

3. Currently the computational benefits are presented in the context of model networks sampling from what might best be described as a prior distribution, as opposed to a posterior that's inferring the correct label for an input image (e.g. for the first two models). The problem with that approach is that the time to transition from one state to another is directly confounded with how close to factorial the distribution is.

[#14]

Indeed, the simulations in Sec. 2.2 and 2.3 use learned priors. However, in later sections (2.6 and 2.7), we explicitly bias the competing interpretations/solutions to model sensory evidence; thus, these experiments show sampling from posteriors. We have edited the text to highlight this point (l. 424, l. 449 and l. 501).

In a purely mathematical sense, there is no strict separation between posteriors and priors. In practice, posteriors are effectively priors with changed biases. Some of the work we cite has already demonstrated spike-based sampling from posterior distributions, e.g., during inference from partially occluded images (Kungl et al., 2019, Frontiers in Neuroscience; Dold et al., 2019, Neural Networks). We also performed additional experiments showing sampling from posterior distributions in our model (see Fig. S5 in the *Supplementary Material* and the response to #15). See also the reply to comment #32, which is related to the issue of priors vs. posteriors.

Regarding the second point, assuming the reviewer refers to completely factorizing distributions over binary variables, i.e., effectively independent neurons, then this directly lines up with our argument that, at high temperatures, the resulting distribution becomes more uniform (and factorizable). This allows the network to more easily change its state and explore the state space, increasing the chance to fall into a different deep mode during the next low-temperature phase. Note that it is at low temperatures that the network samples from the true underlying distribution, which explicitly should not factorize in order to capture non-trivial correlations (e.g., images).

On a related note, we should also point out that the ability to mix is not only a property of the distribution but also of the sampler itself. For example, the synaptic time constants τ_{syn} introduce a lower bound on the ability of the network to mix, independently of the underlying distribution.

As far as I can tell, the authors currently have not quantitatively demonstrated that the oscillations do anything actually useful. The hypothesized benefit e.g. during inference, remains a conjecture. A better and more direct test would be to measure how long it takes for one of their models to infer the correct category for an observed image starting at a random state. That time could be compared with and without the proposed oscillations on the temperature verifying that sampling from the wrong distribution during the phases with high temperature is compensated by the higher speeds at which the correct category is reached/inferred.

[#15]

> To show the benefit of oscillations for inference, i.e., sampling from a posterior distribution, we performed an additional simulation (Fig. S5 in the *Supplementary Material*, see also the response to #32) where a superposition of two different input classes is presented as input, and the model should infer the correct label distribution (the posterior). We found that with constant background input, the posterior does not reproduce the uncertainty of the inputs as one class is strongly favored. Both classes occur roughly equally with oscillations, showing that oscillations benefit sampling from the posterior. This finding also underscores that there is no substantial difference for sampling from prior or posterior distributions (see the response to #14).
>
> Together with the experiments from the main text, this experiment shows that oscillating background input improves sampling from various distributions over diverse data spaces, both with (posterior/inference) and without external evidence (prior). We would argue that these scenarios relate directly to benefits for (biological) agents, in the absence of sensory evidence (dreaming, corresponding to sampling from a prior distribution), and in the presence of sensory evidence (wakefulness, corresponding to sampling from a posterior).

4. The section comparing their proposal to neurophysiological data is incomprehensible to me. The model remains completely unclear even with the information in the Methods, with no intuitions provided for why the model behaves the way it does. It is also unclear which of the modeling findings are robust to the model details and to what degree the discrepancy between model and data presents a challenge for their proposal in general, or just this particular model. I personally would probably omit this section - the key proposal by authors about the role of oscillations is sufficiently abstract that reliably testing it using empirical data requires multiple uncertain links that might go beyond the scope of this paper.

[#16]

> We added a schematic drawing to illustrate the network model (Fig. 7a) and a discussion with an intuitive explanation of the basic behavior of the model (l. 436). We furthermore expanded the explanation of changes in the model behavior depending on its parameters (l. 449) with more details regarding the underlying reasons. To test the robustness of the model, we systematically varied the main model parameters (see Fig. S7 in the *Supplementary Material* and l. 486). We found that the model is relatively robust to parameter variations if the level of inhibition is sufficiently high to allow only one assembly to be active at any point in time.

Exposition:

- The manuscript presents several models without clearly describing any one of them. I think it would help a lot to focus on one model, e.g. the one based on the MNIST dataset, describe the model in detail, as well as clearly demonstrating the effect and benefits of oscillations. The same model could then be implemented using conductance-based LIF neurons without much needed extra explanations.

[#17]

> We have purposefully chosen to present a number of different models in order to highlight the wide applicability of oscillation-induced tempering in spiking networks, as well as its biological plausibility, which we believe to be a manifest strength of our idea. However, we agree that our model descriptions could have been clearer and therefore tried to improve the presentation throughout the manuscript (see, e.g., lines 260, 344 and 449, as well as our responses to points #16, #41 and #45). We also emphasized the sampling from posteriors in Sec. 2.6 and 2.7.

- The current manuscript assumes too much prior knowledge. When building on prior work it would really help to briefly summarize the key result that is being incorporated into the present work.

[#18]

Agreed. We improved the explanation of results from prior work in the revised manuscript (see, for example, lines 106, 135 and 147).

- When describing the modeling results, it's often helpful to move from a individual examples to summary statistics to how summary statistics depend on parameters, not the other way around as currently, e.g. in Fig. 5.

[#19]

We agree. We re-ordered the panels accordingly, with panels a-d now showing specific examples. (Note: this is now Fig. 4.)

Minor:

- Fig 5f: blue distribution impossible to discern: 50% of mass at 2, and 50% at 998?

[#20]

We improved the figure by moving the inset to a separate panel and expanded the corresponding text (l. 783 and l. 788). Yes, the two modes are equally high and correspond to 50% of the total mass; note, however, that the ordinate represents the number of appearances of a particular mode duration, so it is not normalized. (Note: this panel is now Fig. 4b.)

- Fig 5g: does this suggest that the tempered sampler is worse than the factorized one up to 20 cycles? Isn't that a problem?

[#21]

This observation raises an important point. The transient "underperformance" of the tempered sampler represents an artifact of the ISL measure, which tries to quantify image quality and diversity simultaneously. Roughly speaking, the ISL measures how close a collection of images is to all the images in a dataset. A single averaged MNIST image is, by construction, already quite similar to the entire MNIST dataset. In particular, its ISL is higher than for any single, clean MNIST image. So for a small number of cycles, the diversity of the clean images produced by our tempered network is still too low to yield a high similarity to the entire MNIST dataset. This changes as soon as enough modes were covered. We have added a short explanation to the caption, as well as to the corresponding section *Indirect sampling likelihood* in the Methods. (Note: this panel is now Fig. 4g.)

- How does the background noise affect spiking statistics?

[#22]

In general, the answer to this question has two components, and we assume the reviewer refers mostly to the temporal aspect.

First, for a given background noise level, the temporal single-neuron and network statistics are significantly different from independent Poisson. In particular, single neurons can exhibit bursts, and therefore be autocorrelated. Due to lateral interactions, spike trains are also cross-correlated. For a more detailed discussion, we point to Dold et al., 2019, Neural Networks, in particular Sec. 3.1 and 3.2. For the experiments in this manuscript, we only discuss mean firing rates, which are plotted in the new Fig. S1 in the *Supplementary Material* and Fig. 7c.

The second component, of course, relates to the core idea of the manuscript. When the background noise level (i.e., rate) changes, the shape (slope, offset) of single neurons activation functions changes (see Fig. 1c-e), and consequently also the (spatial) network spiking statistics (Fig. 2b,d), which are fully captured by the sampled distribution.

- Fig 6: meaning of columns unclear

[#23]

(Note: this is now Fig. 5.) The four columns represent different background scenarios. We have clarified the meaning in the caption of Fig. 5.

- How does alpha relate to beta relate to temperature?

[#24]

In the current-based case, we have $T \propto 1/\beta$ (Eq. 7), while in the conductance-based case we found that $T \sim \sqrt{\alpha}$. We have tried to accentuate these relationships further in l. 138, l. 147, l. 335 and Eq. 13 of the revised manuscript.

- It would help to more clearly motivate the conductance-based simulations, and how exactly they differ. Currently, applied to different models it is very hard to understand the relevant differences between the two implementations and what they mean with respect to the central claim of the paper.

[#25]

We added more details regarding the motivation for the conductance-based experiments, in particular concerning the differences to the current-based simulations (l. 260). Furthermore, we added a summary of the difference between the current-based and the conductance-based results along with their meaning for the paper's main claim (l. 337).

- Consider using autocorrelation to quantify mixing time

[#26]

Because we trained our networks in Fig. 3 and 4 on distinct images, we chose the mode duration as the most direct measure of the "computational state" of the network. However, we agree that mixing should also become apparent in the autocorrelation. Therefore, we now also evaluated the average autocorrelation of the single neuron's spike probability (see Eq. 36) for the label and visible layers (see Fig. S3 in the *Supplementary Material*; l. 779). The area under the mean autocorrelation function is reduced for the tempered sampler compared to the untempered one, indicating better mixing, in agreement with the results obtained through the mode duration analysis.

- Some of the results (e.g. in Fig 5) are presented in terms of cycles, but the results must depend on the frequency of the tempering relative to the biophysical time constants in their spiking model, right? Please make that explicit.

[#27]

We clarified the discussion of this aspect in the revised text (l. 553).

- Fig 5: What is DKL(I)? Shouldn't there be two distributions in the argument?

[#28]

We agree that this notation should be improved. We removed "(I)" from the axis label and clarified in the revised caption that it is the DKL of the label layer activation with respect to the uniform distribution.

- P.10: Typo "Although νexc = νinh in this case (Fig. 6a, first column), .." should be Fig.6c

[#29]

Fixed.

- What is the motivation for Eqn. 10?

[#30]

We added a brief discussion of the motivation for choosing this form (l. 318).

Reviewer #3

- The authors present a theory on the way recurrent neural networks can perform stochastic computations. In particular, the authors argue that approximate Bayesian computations can be efficiently performed if oscillations are present. The key to their argument is that in order to perform efficient inference sequential samples need to be obtained from a probability distribution (notably, the posterior distribution) and a faithful representation of such a distribution requires fast mixing, i.e. that all possible corners of the state space with finite probability can be visited without being trapped in a particular local mode of the distribution. The starting point of the paper is that a background network of neurons imposes a dynamics on a set of 'signal neurons' that stochastically explores the activity space, thus implicitly defining a 'distribution' of activities. The authors then provide an elegant argument that under some circumstances the entropy of this distribution can be simply and systematically decreased and thus achieve fast mixing. A minor note here concerns readability: while the argument can be understood from the text, the flow of the text is not linear and bits and pieces of the line of thoughts need to be gathered by going back and forth in the text. The text would benefit from a clear cartoon that walk the reader through the individual steps.

[#31]

We added an extensive introduction to the Experiments and Results section that gives an overview of the steps and main results in the respective subsections, helping to guide the reader through the paper's main arguments (l. 74).

- The theory behind controlling the width of the activity distribution provides some interesting insights into network dynamics. However, several critical questions regarding the role of such a process in approximate inference remained elusive. First, the authors start with the argument that the nervous system needs to be able to represent uncertainty. Representing uncertainty in terms of the variability of neural activity has gained support in recent years. Here, uncertainty affects the entropy of the distribution therefore it would be critical to see if the variability expressed by the network can be related to uncertainty. Critically, increased uncertainty has similar effects to the 'annealing' process proposed here. It would therefore be necessary to demonstrate that the two components of the algorithm (representing uncertainty by neural variability and performing annealing) can be achieved using the same substrate. In general, a more principled link between the distribution defined by the recurrent connections and the posterior distribution would be important.

[#32]

We agree that a more explicit discussion of uncertainty is in order. To clarify this point, we performed an additional experiment which is summarized in Fig. S5 in the *Supplementary Material* and added a paragraph to the Discussion (l. 501), which, we hope, addresses the raised issues.

Note that uncertainty is represented in our model through the diversity of samples obtained at different time points during the sampling process. In particular, when samples at time points of the oscillation with temperature $T = 1$ are considered, these samples are drawn from the represented distribution. In Fig. S5 in the *Supplementary Material*, we provided our network with sensory (visual) evidence by clamping the upper part of its visible layer to an ambiguous MNIST image that could be interpreted as either an 8 or a 9 (panel d). The uncertainty of the posterior over (compatible) labels can now be read out by observing the activity in the label layer (see Fig. 3a for network structure and label units) at various time points during the sampling process where the temperature is $T = 1$. The label counts are shown in Fig. S5 in the *Supplementary Material*, panel e. The red bars show the counts for background oscillations. The network's uncertainty is almost completely limited to the correct compatible labels 8 and 9 (and much less likely to the labels 3 and 2). Without oscillations (blue), the network is almost constantly stuck in one interpretation; its certainty about the underlying truth is thus higher, but wrongly so, because the evidence was explicitly constructed to favor an 8 and a 9 equally. This shows that the model is able to represent uncertainties (through sampling) while being tempered and can do this more efficiently, i.e., with fewer samples than in the untempered case. We also refer to our replies to comments #14 and #15, which are also related to the issue of priors vs. posteriors.

- Second, the main focus of the paper is the speeding up of the faithful representation of a probability distribution through sampling. Annealing is indeed a well-established method in statistics to achieve this goal. The proposed format, however, poses a number of problems that remain unaddressed in the paper. First, oscillatory annealing leaves only a narrow window left to read out the true distribution. That is, considering the only discussed frequency range, theta activity, a fraction of the 100-ms cycle open for sampling the represented distribution. While the paper provides insights into how the oscillation can achieve mixing between modes but the representation of the uncertainty is left open. In a population of neurons a single sample could be obtained in a much shorter time frame than the cycle of theta activity. The cycle of theta activity corresponds to the perceptual time scale, therefore pruning samples by using the oscillation can be detrimental to perception. In sum, it would be important that the representation of uncertainty is not hurt by introducing annealing.

[#33]

This paper focuses on probability distributions with distinct and deep modes, where mixing is hard. While a sampling method without oscillations could, in principle, obtain a first sample (or a few samples) faster than one oscillatory cycle, these samples would provide a skewed (unimodal) representation of the underlying (multimodal) distribution. Even worse, they might lie in narrow local minima that correspond to none of the major modes (which is why sampling methods usually require a certain burn-in time to "forget" their initial state). As we show in, e.g., Fig. 3 or Fig. 4, even if a first mode can be reached, it would be hard to reach different modes without oscillations subsequently.

The time window in which computational results can be read is indeed only a restricted part of the entire cycle (see Fig. 6c and Fig. S2 in the *Supplementary Material* which provides quantitative data in the

form of the KL-divergence over a cycle). We propose that this separation of computations into different phases provides a benefit by making it explicit when a computational result can be read out (l 237). It is, however, important to note that the time window depends amongst others on the exact time course of the background input (see also Fig. S4 in the *Supplementary Material*). We used (for simplicity) sinusoidal background input modulations, but different forms of cyclic activity, or, more generally, any form of alternating background input phases, could provide the same computational benefit. In the simplest case, there could be two alternating phases of (constant) high and low activity – corresponding, for example, to cortical UP and DOWN states – providing high and low-temperature phases (see also the response to #12). In this case, the readout phase could be effectively as long as the phases themselves. We added a discussion of this point to the text (l. 640 and l. 656).

- Several studies have previously addressed the question of efficient sampling in neural networks. These rely on techniques that are widespread in machine learning, e.g. Hamiltonian Monte Carlo is core to solutions in probabilistic programming. Unfortunately, the current paper does not build and does not critically review these alternatives. The most direct comparison directly addresses decorrelation of samples in a neural network using balanced neural networks (Guillaume Hennequin, Laurence Aitchison, Mate Lengyel, Fast Sampling-Based Inference in Balanced Neuronal Networks, Advances in Neural Information Processing Systems 27 (NIPS 2014)). This paper has clear parallels in the network structure, objectives, and assumptions with the current paper. The other paper, which is cited but not contrasted with the current proposal is 'The Hamiltonian Brain: Efficient Probabilistic Inference with Excitatory-Inhibitory Neural Circuit Dynamics'. This paper uses Hamiltonian Monte Carlo a sampling method that excels in helping mixing. Here the role of inhibitory neurons and oscillations is complementary to the current proposal. It would be a natural testbed to contrast the effectiveness of HMC with tempering in overcoming sampling a multimodal distribution.

[#34]

Yes, also here we agree that a more detailed discussion would benefit the manuscript. Most importantly, Langevin (Hennequin et al., 2014) and HMC (Aitchison and Lengyel, 2016) sampling are based on continuous-value distributions. The proposed network models thus use rate-based approximations to arrive at a continuous-value activity. On the other hand, our model uses discrete random variables that relate directly to neuronal spiking. We are not aware of how to perform HMC in a spiking model; thus, we cannot directly compare the two approaches. We have added a corresponding paragraph to the Discussion (l. 539).

Minor:

- The narrative of the results would benefit from cleaning. Linearity of the argument is hurt a number of times and motivations of the steps are not sufficiently clearly spelled out.

[#35]

We address these issues in the revised manuscript, for example, in Sec. 2.2. See also #31 above, #37 below, and similar points raised by the other reviewers (#7, #17-19 and #25) and the responses to those.

- Similarly, the introduction section could benefit from a clarification (e.g. sampling at the end of the first paragraph is introduced without much background given, and the paper immediately navigates to 'mixing' a term that is quite complex and requires more explanation).

[#36]

Agreed. We expanded this part of the Introduction to clarify the mixing problem (see l. 37 and l. 43).

- The sentence on p 5 'Thus, oscillatory background activity can be interpreted as tempering, a periodic cycle of heating and cooling, with hot phases for mixing and cold phases for reading out the most relevant samples of the correct distribution.' is little motivated: there is a leap in the argument that is hard to follow, even though the insight is later demonstrated.

[#37]

Agreed. We added more explanations before introducing the notion of tempering (l. 167). (See also our reply to #31).

- 'In doing so, we unify two individually well-studied, but previously unlinked aspects of cortical dynamics under a common normative framework: probabilistic inference and cortical oscillations' — please specify, as note above this claim is not true

[#38]

We thank the reviewer for pointing this out. We revised the text to clarify that we specifically address the role of spikes (l. 43). (See also our reply to #3).

- The penultimate paragraph of the introduction ventures towards topics that are not addressed in the results but provide a perspective to the current work, I suggest moving those to the discussion.

[#39]

Good point, done (l. 565.).

- Sampling the troughs of oscillation is proposed to obtain decorrelated samples the target distribution. It would be insightful to see an analysis on the width of the time windows that correspond to a faithful representation of the target distribution.

[#40]

We quantified the mismatch between the network states and the target distribution using the KL divergence over the oscillatory cycle in Fig. S2 in the *Supplementary Material*. As expected, we found that mismatch is small when the background-induced temperature is close to the reference temperature $T = 1$; for too high or too low temperatures, the mismatch increases. However, it is important to note that the time window(s) for accurate matching depends on two factors. First, it depends on the underlying distribution; for the MNIST network with its deep modes, stable and distinct labels can be seen over wide time windows (see Fig. S4 in the *Supplementary Material*). Second, it depends on the time course of the background modulation – see also #33 and l. 656 for a discussion of this point.

- The motivation and details of particular networks used during the results is little motivated it is very hard to keep pace with the manuscript at places where these models are introduced.

[#41]

We tried to remedy this shortcoming by expanding the corresponding parts of the paper to make the motivation for and behavior of the different models clearer (l. 260 and l. 424, see also #17).

- Also, the link between the text body and the figures is often weak and interpreting figures is not straightforward (a prime example is Fig 3a).

[#42]

Agreed. (Note: what was previously Fig. 3 is now Fig. 2.) We have expanded the references to the figures in the main text (see l. 124 to 130 for the discussion of Fig. 2). See also our reply to #7 above.

- Similarly, on Fig 4 it is hard to understand why the particular distribution is relevant and why are there multiple modes?

[#43]

(Note: this is now Fig. 3.)
The different modes represent the NORB dataset's different image classes (truck, van, hippo, etc.). We chose this dataset because we think it illustrates the transition from cold to hot phases particularly nicely. Aside from that, we could, of course, choose any distribution, and it would show similar behavior (cf. Fig. 2). We clarified the motivation behind the choice of the distributions and the emergence of the modes in l. 74, l. 188, and l. 193.

- Further, in section 2.4 @ Eq. 9 the motivation for the current form is not clearly provided although reading through the section will yield an understanding.

[#44]

We added some clarifications regarding the motivation of the equation (l. 280 to l. 285).

- Same for the network architecture in 2.5: very brief descriptions are provided and choices are not sufficiently justified.

[#45]

We added a more detailed description of the network model, including motivation for this model, to the text (l. 344). We furthermore expanded the description of the network architecture in Sec. 2.6 (l. 379).

- Fig 6a,b: a clearer reference to the panels would be useful.

[#46]

(Note this is now Fig. 5a,b.)
We improved the clarity of the figure caption regarding these panels and their references in the text (l. 270).

- No direct consequences of the proposal has been addressed in the paper that could contrast it with empirical data. For instance, the lower temperature samples would introduce slower decorrelation of spikes, while higher temperature would introduce faster decorrelation.

[#47]

Indeed, this intuition is correct at both a microscopic (neuronal activity) and macroscopic level (perception, behavior). Following this suggestion and those of the other reviewers (cf. #15, 25), we added a comparison of autocorrelation times between static and oscillatory background (see Fig. S3 in the *Supplementary Material*). At a more macroscopic level, oscillatory background input would influence the frequency of perceptual switches. For example, for multi-stable or incomplete images such as those in Fig. S5 in the *Supplementary Material*, perceptual switches should happen in cortical up-states, as measured, for example, by EEG. We would thus predict a monotonic dependence of the switching frequency of perceptual representations on the switching frequency of up and down states. Furthermore, we clarify how the experiments relating to Jezek et al. (2011) offer a contrast to experimental data. We added the corresponding paragraph to the Discussion (l. 621).

- The empirical data that is presented refers to the ambiguity of place cell representations at different phases of theta. An influential account of place cell activity that has strong ties to functional models claims that place cell activity at different phases of theta oscillations correspond to places at different parts of the animal's movement trajectory (Brad E. Pfeiffer & David J. Foster, Hippocampal place-cell sequences depict future paths to remembered goals, Nature 497:74–79 (2013)). Since this is the one experimental outlook the results section provides, it would be useful for the reader to contrast the explanatory power of the competing theories.

[#48]

This is a good point. However, we think that the view taken in Pfeiffer and Foster is consistent with our interpretation of a sampling strategy on the level of trajectories (temporal sequences) rather than static values. We extended the corresponding part of the Discussion (l. 621), See also the replies to #12, 33, 40, and 47.
