# Supplementary material for: Cortical oscillations support sampling-based computations in spiking neural networks
Source: PLoS Comput Biol. 2022 Mar 24;18(3):e1009753. doi: 10.1371/journal.pcbi.1009753 (PMC8947809; doi:10.1371/journal.pcbi.1009753)
Supplement: Attachment — Submitted filename: response-to-the-reviewers.pdf [file pcbi.1009753.s010.pdf]

Author response to reviewer comments

We thank the reviewers again for their very constructive remarks and interesting questions. Below, we address the remaining questions from Reviewer 3.

Reviewer #3

At issue #32 the authors refer to a newly added figure. It is indeed true that multiple interpretations to the same stimulus greatly contribute to uncertainty and this aspect is covered in this analysis. What my comment was referring to is the issue of another form of uncertainty that plagues everyday inference: when a single-mode posterior becomes wider as a result of poorer data (contrast, limited observation, occlusion, etc). This form of uncertainty results in a similar form of widening of the posterior as annealing does. I believe that it is crucial property of sampling that such changes in the posterior can be reflected. The current phrasing of the manuscript suggests that the proposed method offers a full-feldged solution for sampling the posterior but the actual focus is much narrower since the above widening is not covered at all. I believe that covering the concept of such posterior widening would be essential for the readers to have the scope and limitations of the paper.

[#1]

> We believe that our model does address the issue raised by the reviewer here, but we should be careful about differentiating between two phenomena: 1) the widening of individual modes due to decreasing certainty of the evidence (e.g., through weaker, noisier, or partially occluded sensory input) and 2) the widening of individual modes due to a rise in temperature.
> 1) Uncertainty resulting from poorer data is well captured by our sampling model, which by construction samples from a posterior as a function of the evidence. For example, we can choose a high-contrast "6" as a visual input (i.e., strong evidence) and then slowly reduce the contrast (weaker evidence corresponding to a wider local mode). The visible layer will initially sample from states that are very close to the input, but as the stimulus weakens, the network will start exploring a wider range of options within the same mode and produce, for example, different styles of the written digit "6". Something similar can be seen in Fig. S4 between 18 s and 24 s; while there it is not a consequence of decreasing evidence, it nevertheless illustrates how the scenario above would take place.
> 2) Increasing temperature does broaden local modes, but in a way that does not (and should not) interact with the evidence. This is because correct samples are only read out at temperatures close to the reference temperature (by construction, $T \approx 1$). At that temperature, the correct distribution is sampled, which can change depending on the available evidence, as discussed above.

At issue #33 the authors propose that up/down states can provide additional opportunity for an annealing-like behavior. I find this proposal intriguing, especially because this phenomenon has a wide literature, including papers that feature intracellular recordings (e.g. (Tan, A. Y. Y., Chen, Y., Scholl, B., Seidemann, E., & Priebe, N. J. (2014). Sensory stimulation shifts visual cortex from synchronous to asynchronous states. Nature, 509(7499), 226229. http://doi.org/10.1038/nature13159), that can provide the necessary means to test the theory. I find it thought provoking that the paper provides links to a number of phenomena but this links remains at the speculative side despite available data.

[#2]

> We agree with the reviewer and emphasized the connection to data from Engel et al. (2016), where it is shown that monkeys perform better at recognizing a change in their visual field if it occurs during an on-state. This is compatible with our prediction that during an off-state (low background) networks are largely bound to a local mode and literally stick to their current belief in a Bayesian sense. To detect a subtle change in their input requires them to be able to change their belief, which is more likely to happen when the probability landscape is flatter, i.e., at high temperatures, which correspond to high background rates and thereby to cortical on-states, as observed by Engel et al. We expanded the discussion on this point in l. 644-653.

At issue #48: I am not sure I understand the argument the authors provide. According to the proposed role of oscillations, a neuron population is sampling the same distribution at different phases of the oscillation but the temperature of this distribution varies with the phase of the oscillation. The work on the Foster lab (also Loren Franks and David Redishs labs) points out that at different phases of the theta oscillation different portions of the trajectory are sampled that correspond to past present and future locations of the animal. It is hard reconcile this view with the idea that the same distribution is sampled at different phases. Since hippocampal theta is the only

point where the paper ventures into actual comparison with experimental data, I believe that clarifying this issue is important.

[#3]

As discussed by Habenschuss et al. (2013, PLoS CB), sampling models can be extended to incorporate a phase-dependent distribution based on external input, giving rise to the sampling of sequences, as described by the data referenced by the reviewer. We have clarified this aspect in the discussion (l. 629). We stress again that our model is not intended as a model of (all aspects of) spatial processing in the hippocampal formation, but we chose to include the final experiment as a proof of concept providing inspiration for possible experimental verification of the main aspect of our proposal.
